# Association Between Serum Retinol and α-Tocopherol Levels and Metabolic Syndrome in Korean General Population: Analysis of Population-Based Nationally Representative Data

**DOI:** 10.3390/nu12061689

**Published:** 2020-06-05

**Authors:** Taeyun Kim, Jihun Kang

**Affiliations:** 1 Department of Internal Medicine, The Armed Forces Goyang Hospital, Goyang-si 10267, Gyeonggi-Do, Korea; jimsb89@naver.com; 2 Department of Family Medicine, Kosin University Gospel Hospital, Kosin University College of Medicine, Busan 49267, Korea

**Keywords:** metabolic syndrome, retinol, α-tocopherol, KNHANES

## Abstract

This study aimed to evaluate the association between serum retinol and α-tocopherol levels and metabolic syndrome (MetS) using data from the Korea National Health and Nutrition Examination Survey. Of the 24,269 individuals, 5885 adults (2672 men and 3213 women) were included. The prevalence of MetS and its components according to quartiles of serum retinol and α-tocopherol levels was calculated. Multivariate linear regression model was used to calculate the number of metabolic components according to serum vitamin levels. The association between serum vitamin levels and MetS with its components was assessed using multivariate logistic regression model. The prevalence of MetS was highest in Q4 and lowest in Q1 for both vitamins, regardless of sex. A dose-dependent association between serum retinol and α-tocopherol levels and MetS was observed. After adjustment for covariates, the odds ratio (OR) for MetS in Q4 compared to Q1 was 2.351 (95% CI: 1.748–3.163, *P*_trend_ < 0.001) in the retinol group and 2.559 (95% CI: 1.953–3.353, *P*_trend_ < 0.001) in α-tocopherol group. Among metabolic components, hypertriglyceridemia, high fasting glucose, and high blood pressure was positively associated with serum retinol and α-tocopherol levels. In conclusion, high serum retinol and α-tocopherol levels were associated with increased risk of MetS.

## 1. Introduction

Metabolic syndrome (MetS) refers to a cluster of medical conditions including hypertriglyceridemia, low level of high density lipoprotein (HDL) cholesterol, hypertension, impaired fasting glucose, and central obesity simultaneously [1]. MetS is not only an important determinant for diabetes, cardiovascular disease, and cancer, but also increases overall mortality [2,3,4]. Globally, the prevalence of MetS reached nearly 20% among adults, and this number is continuously increasing [5]. In Korea, the prevalence of MetS increased from 24.9% in 1998 to 30.5% in 2013, thus, and MetS is considered an emerging public health issue [6,7].

Although the pathophysiological mechanisms involved in MetS are intricate, central obesity and insulin resistance (IR) are widely accepted as key etiological factors of MetS [8]. In addition, several factors such as age, alcohol consumption, smoking, and physical inactivity are associated with increased IR and risk of MetS [9,10,11,12]. 

Serum retinol and α-tocopherol are lipid soluble vitamins and play important roles in vision, immune system, and cell protection [13]. However, recent studies implied that these micronutrients may have negative impact on health. Retinol binding protein (RBP), which maintains serum vitamin A level, is associated with increased IR [14]., body mass index (BMI), triglyceride (TG) level, and blood pressure [15]. A mendelian randomization study found similar results showing that the number of RBP-4 variants were associated with increased risk of hypertriglyceridemia [16]. In addition, an earlier cross-sectional study from a group in Germany showed that higher serum vitamin E level was associated with increased visceral adipose tissue and risk of MetS [17]. However, another previous study reported an inverse association between vitamin E consumption and MetS [18]. Consumption of oral vitamin E for three months showed transient improvement in IR [18]. 

However, the effects of serum vitamins A and E on MetS have not been fully elucidated, especially in Asian populations. Furthermore, because most studies were based on questionnaires, only a few studies have assessed serum vitamin levels in relation to MetS. Therefore, the present study aimed to evaluate the association between serum vitamin levels and MetS using the nationally representative data from the 7th Korean National Health and Nutrition Examination Surveys (KNHANES).

## 2. Materials and Methods

### 2.1. Study Participants

The dataset from the 7th KNHANES (2016 to 2018) was used in the present study. The detailed protocols for the survey were presented in a previous study [19]. Briefly, KNHANES is a population-based, cross-sectional health and nutritional survey conducted annually by the Division of Chronic Disease Surveillance under the Korea Centers for Disease Control and Prevention and the Korean Ministry of Health and Welfare. The sampling plan followed a multi-stage clustered probability design, and sample weights were constructed based on the population census of Korea to obtain a national representative sample. The dataset from KNHANES is available online to the public at the Korea Centers for Disease Control and Prevention website.

Among the 31,689 eligible individuals, 24,269 subjects participated in the 7th KNHANES with a response rate of 76.6%. Participants were selected with a proportional allocation system sampling with multistage stratification based on age, sex, and geographical area. KNHANES includes laboratory results, physical examination results, health-related interviews, and nutritional questionnaires.

Of the 24,269 individuals, those who were younger than 19 years (n = 5956), those without full information regarding components of metabolic syndrome (n = 743), those without data on serum retinol and α-tocopherol levels (n = 11,241), and those without data on other variables of interest (residence, household income, education, smoking status, alcohol consumption, physical activity, and high sensitivity C-reactive protein [hs-CRP], n = 444) were excluded. Finally, 5885 individuals (2672 men and 3213 women) were included in the analysis.

### 2.2. Ethical Approval

The study protocol was approved by the Institutional Review Board (IRB) of the Kosin University Gospel Hospital (No. 2020–03–053), and the study was conducted according to the Declaration of Helsinki. All study procedures were in accordance with STROBE guidelines. Written informed consent was obtained from all individuals before participating in the survey. 

### 2.3. Data Collection and Measurements

MetS was defined in accordance with the National Cholesterol Education Program Adult Treatment Panel-III [20], and abdominal obesity was defined based on the criteria of the Korean Society for the Study of Obesity [21]. MetS was diagnosed when three or more of the five following factors were present: (a) WC ≥ 90 cm for men and ≥85 cm for women, (b) TG ≥ 150 mg/dL or current medication for high TG, (c) HDL-cholesterol < 40 mg/dL for men and <50 mg/dL for women or current medication for low HDL-cholesterol, (d) fasting glucose ≥ 100 mg/dL or current medication for diabetes, and (e) systolic blood pressure (SBP) ≥ 130 mmHg or diastolic blood pressure (DBP) ≥ 85 mmHg or current medication for high blood pressure. 

All laboratory samples were acquired by skilled medical assistants and transported to the Central Laboratory (NEODIN Medical Institute, Seoul, South Korea). TG level, HDL-cholesterol level, and fasting glucose level were measured with the enzymatic method, homogeneous enzymatic colorimetric method, and Hexokinase UV method, respectively, with a Hitachi Automatic Analyzer 7600–210 (Hitachi, Japan). In addition, accuracy of HDL-cholesterol level was further enhanced through standardization according to National Institute of Standards and Technology. Serum retinol and α-tocopherol levels were measured with high-performance liquid chromatography-fluorescence with Agilent 1200 (Agilent Technologies, Santa Clara, USA) using the Chromsystems (Munich, Germany) reagent kit. Hs-CRP level was measured with immunoturbidimetry using Cobas (Roche Diagnostics, Basel, Switzerland).

Data on sociodemographic characteristics including age, sex, residence, household income, education, smoking status, alcohol consumption, and physical activity were acquired with the help of self-administered questionnaires or face-to-face interviews. Residential area was categorized into rural and urban. Educational level was divided into three groups: middle school or lower, high school, and college or higher. Household income level was calculated as the average income of all family members and divided into quartiles. Smoking status was categorized into never, former, and current smokers based on the Centers for Disease Control and Prevention classification [22]. Never smoker was defined as someone who had ever consumed less than 100 cigarettes or does not currently smoke. Former smoker was defined as someone who had ever consumed more than 100 cigarettes in the past but does not currently smoke. Current smoker was defined as someone who consumed more than 100 cigarettes and is still smoking. Alcohol consumption status was categorized into three groups: non-consumers, high-risk consumption more than once per week, and low-risk consumption less than once per week. High-risk alcohol consumption was defined as 7 (alcohol 60 g) or more drinks for men and 5 (alcohol 40 g) or more drinks for women [23]. Adequate physical activity was defined when one of the three following criteria was satisfied: (a) at least 150–300 min per week of moderate intensity exercise, (b) 75–150 min per week of vigorous-intensity physical activity, or (c) equivalent combination of moderate- and vigorous-intensity aerobic activity.

Dietary data were collected using 24-h recall method by face-to-face interview. Based on Korean Dietary Reference Intakes for Koreans, dietary intake amount of vitamin A was measured with the use of retinol equivalent (RE) and derived from the following formulation: µg RE = µg retinol + µg β-carotenes/6. However, data regarding vitamin E intake were not available in the 7th KNHANES database.

Anthropometric measurements were performed by trained survey assistants. BMI was calculated as body weight (kg) divided by the squared height (m^2^). Height was measured to the nearest 0.1 cm and weight was measured to the nearest 0.1 kg. BMI was categorized based on the Korean Society for the Study of Obesity guidelines [21].: underweight (<18.5 kg/m^2^), normal (18.5–22.9 kg/m^2^), pre-obese (23–24.9 kg/m^2^), and obese (≥25 kg/m^2^).

### 2.4. Statistical Analysis

Because the KNHANES data were produced using multistage, stratified, and probability sampling, all statistical analyses were performed under complex sample analyses in SPSS. Sampling weights were applied for study participants to represent the Korean population. 

Categorical variables (residence, household income, educational level, alcohol consumption, smoking status, physical activity, and BMI) were presented as weighted percentages with standard error (SE). Continuous variables (age, hs-CRP, retinol, and α-tocopherol) were presented as mean value with SE and median value with interquartile range (IQR). Metabolic variables (WC, TG, HDL-cholesterol, fasting glucose, SBP, and DBP) were presented as mean value with SE.

Prevalence of MetS and its components was calculated according to serum vitamin levels. Serum retinol and α-tocopherol levels were divided into quartiles—Q1 (lowest), Q2 (lower middle), Q3 (higher middle), and Q4 (highest). The association between serum retinol or α-tocopherol levels and the number of metabolic components was estimated with the linear regression model. Binary logistic regression model was used to evaluate the association between serum retinol or α-tocopherol level and MetS and its components. In multivariate-adjusted logistic regression model, age, sex, residence, household income, education, alcohol consumption, smoking status, physical activity, hs-CRP, and BMI (or not) were applied. The association between serum vitamin levels and MetS was measured by calculating the odds ratio (OR) with 95% confidence interval (CI). Subgroup analyses were performed to identify differences between men and women and to evaluate the association between dietary vitamin A intake and the prevalence and risk of MetS

All statistical analyses were performed with SPSS (version 24 for Windows, Chicago, IL, USA). For all analyses, a *p*-value < 0.05 was considered statistically significant.

## 3. Results

The weighted prevalence rate of MetS was 24.2% (27.4% in men and 21.6% in women). Characteristics of study participants are presented in Table 1. Mean retinol levels were higher in men than women, whereas mean α-tocopherol levels were higher in women than in men. Mean WC, TG, fasting glucose, SBP, and DBP were greater in men than in women. Mean HDL-cholesterol level was greater in women than in men. Men had higher income, education, alcohol consumption, smoking, BMI, and hs-CRP level than women. Women had higher physical activity level than men. 

**Figure 1:**
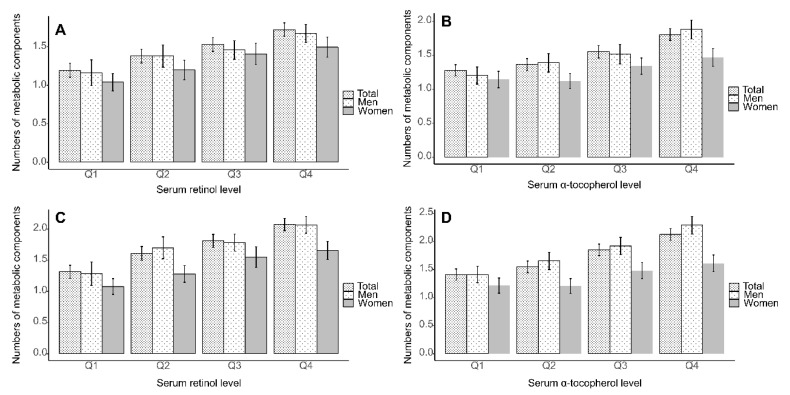
Estimated number of metabolic components with 95% confidence interval according to quartiles of serum retinol and α-tocopherol levels based on multivariate-adjusted linear regression analysis is shown. (**A**) retinol, and (**B**) α-tocopherol with adjustment for body mass index (BMI). (**C**) retinol and (**D**) α-tocopherol without adjustment for BMI (*P*_trend_ < 0.001 for all analyses). Multivariate-adjusted model included age, sex, residence, household income, education, alcohol consumption, smoking status, physical activity, hs-CRP, and BMI (or not) as covariates. Hs-CRP, high sensitivity C-reactive protein; BMI, body mass index.

The number of metabolic components according to serum retinol and α-tocopherol levels is presented in Figure 1. Individuals in Q4 of serum retinol and α-tocopherol levels showed the highest number of metabolic components, regardless of sex.

Table 2 shows the weighted prevalence and OR for MetS according to quartiles of serum retinol and α-tocopherol levels. Prevalence of MetS was high in the order of Q4, Q3, Q2, and Q1 in both retinol and α-tocopherol. According to the multivariate-adjusted model, ORs for MetS were positively associated with retinol (*P*_trend_ < 0.001) and α-tocopherol (*P*_trend_ < 0.001) in a dose-dependent fashion, regardless of BMI adjustment. OR for MetS in the highest quartile of retinol was 2.351 (95% CI: 1.748–3.163) and in the highest quartile of α-tocopherol was 2.559 (95% CI: 1.953–3.353) compared to lowest quartiles of respective vitamin levels

Multivariate-adjusted ORs for the components of MetS (abdominal obesity, high TG, low HDL-cholesterol, high fasting glucose, and high blood pressure) according to quartiles of serum retinol and α-tocopherol levels are presented in Table 3. ORs for abdominal obesity, high TG, high fasting glucose, and high blood pressure increased with higher serum retinol (*P*_trend_ < 0.001) and α-tocopherol (*P*_trend_ < 0.001) levels, in multivariate-adjusted models not including BMI. However, with respect to abdominal obesity, statistical significance did not persist after adjustment for BMI.

Unweighted prevalence of MetS and its components according to serum retinol and α-tocopherol levels is presented in Table S1 and analysis without sampling weight did not alter the observed association. Subgroup analyses of the weighted prevalence and ORs for MetS according to the quartiles of serum retinol and α-tocopherol levels are presented in Tables S2 and S3. The prevalence and risk of MetS were positively associated with serum retinol and α-tocopherol levels in both sexes. The relationship between dietary vitamin A intake and MetS is presented in Table S4. A dose-dependent relationship was observed only in male, with multivariate adjustment (not for BMI).

## 4. Discussions

The present study investigated the association between serum vitamin A (retinol) and E (α-tocopherol) levels and the risk of MetS in Korean adults. Our study revealed that both retinol and α-tocopherol levels were associated with increased risk of MetS in a dose-dependent manner, irrespective of sex. Among metabolic components, abdominal obesity, high TG level, high fasting glucose level, and high blood pressure were positively associated with serum retinol and α-tocopherol levels. In addition, when BMI was adjusted for, significance for abdominal obesity was nullified for both sexes, which is more likely due to the high relevance between abdominal obesity and BMI [24]. 

These results are in line with a previous cohort study showing that serum RBP level, which constitutes a good surrogate marker for serum vitamin A level [25], was associated with increased risk of hypertriglyceridemia, high blood pressure, and low HDL-cholesterol level [26]. In addition, Wessel et al. provided similar results showing that plasma retinol and RBP levels were related to elevated risk of hypertriglyceridemia, dyslipidemia, and MetS [27]. Another study showed that α-tocopherol level was positively associated with BMI, WC, and waist-to hip ratio [28]. Moreover, a mendelian randomization study investigating the functions of RBP-4 variants confirmed that plasma RBP-4 level was significantly associated with several metabolic parameters such as hypertriglyceridemia, abdominal obesity, hypertension, and low HDL-cholesterol [16]. In contrast, Park et al. showed that higher vitamin A intake was associated with lower risk of MetS only in Korean women [29].

Previous analyses surrounding serum levels and oral intake of vitamins in the Korean population have provided conflicting information. In a study including 404 Korean adults aged 60 years or older, Kim et al. showed an inverse relationship in elderly women between oral and serum vitamins (retinol and α-tocopherol) and the risk of MetS [30]. Similarly, higher vitamin E intake has been associated with a lower prevalence of MetS [31]. On the contrary, Cho et al. confirmed that high serum retinol and α-tocopherol levels tended to increase the risk of MetS, in a much smaller study sample [32]. Although the reason for these discrepant findings is unclear, differences in age groups (e.g., general population vs. only elderly), sex, adjusted compounders, and study designs could be attributable for this inconsistency.

The present study evaluated the status of vitamin A and E by measuring serum retinol and α-tocopherol concentration, respectively. Retinol is the most commonly used index to evaluate vitamin A status, and its reliability is documented in previous studies [33]. Most studies evaluating vitamin E status and Recommended Dietary Allowance for vitamin E are based on plasma α-tocopherol concentration and daily α-tocopherol intake amount [34]. Biochemical analysis is a more accurate way to assess vitamin status than questionnaires, although integrating both laboratory sampling and diet surveys would be optimal. In the present study, serum retinol was weakly correlated with oral retinol intake (Pearson’s correlation coefficients = 0.027, *p*-value = 0.049, Figure S1). However, another Korean study failed to show a significant correlation between serum and oral retinol [35], implying multiple factors outside of oral intake, such as the use of nutrient supplements, types of vitamins consumed, methods of food intake survey (24 h recall method or food frequency questionnaires), race, and age could alter serum retinol levels [36]. Furthermore, a previous study investigating the correlation between dietary vitamin intake and serum concentration suggested that multiple independent measurements may be required to control intra- to interindividual variance [37].

Several mechanisms regarding the adverse effects of vitamin A and vitamin A-related parameters on metabolic health have been proposed. First, retinol may induce oxidative stress and modulate activities of antioxidative processes. An in vitro study demonstrated that retinol supplementation caused activation of catalase, glutathione peroxidase, and superoxide dismutase [38]. Second, vitamin A may be an important regulator of β-oxidation activity, which is an important causative factor for MetS [39]. One animal model showed that retinoic acid receptor-deficient mice presented lower mitochondrial β-oxidation activity of fatty acids than wild-type mice [40]. In addition, an experimental study, which revealed that the vitamin A derivate retinoic acid stimulated mitochondrial β-oxidation in human hepatocytes, supports at least partially, these findings [41]. 

With regards to vitamin E, there are several plausible explanations. First, the association between serum α-tocopherol level and adipose tissue mass may be related to this association. Visceral adipose tissue mass assessed by magnetic resonance imaging was positively associated with α-tocopherol/cholesterol ratio, and this ratio was related with increased OR for MetS [17]. Second, delayed excretion of α-tocopherol in patients with MetS could possibly account for this association. The α-tocopherol metabolite levels in urine and blood samples were significantly lower in adults with MetS than in healthy adults, implicating that α-tocopherol catabolism is significantly delayed in patients with MetS [42].

Although meaningful results are shown in the current study, several limitations should be noted. First, considering that cross-sectional studies do not include temporal analyses, the observed association between MetS and serum vitamin level should be interpreted carefully. Second, only serum retinol and α-tocopherol levels were measured; it is possible that other subtypes of vitamin A and E impact metabolic syndrome and its components in different ways. Third, a comprehensive comparison between the dietary intake of vitamins, serum vitamin concentrations, and the risk of MetS was only conducted in retinol, due to the limited information regarding vitamin E intake in the 7th KNHANES. Despite these limitations, the present study robustly estimated the independent association between serum retinol or α-tocopherol levels and MetS after adjustment for potential confounders using nationally representative samples.

## 5. Conclusions

In summary, we observed positive associations between serum vitamin A (retinol) or E (α-tocopherol) and the risk of MetS. The risks of abdominal obesity, high TG level, high fasting glucose level, and high blood pressure significantly increased as the serum retinol and α-tocopherol levels were elevated. Excess serum vitamin levels may have putative harmful effects. Further investigation regarding the longitudinal relationship between serum vitamin levels and metabolic abnormalities is warranted.

## Figures and Tables

**Table 1:** Characteristics of study participants according to sex.

	Total (n = 5885)	Men (n = 2672)	Women (n = 3213)
**Age, years**	47.1 (0.3)	46.2 (0.4)	48.1 (0.4)
**Residence**			
Rural	87.1 (1.3)	87.1 (1.4)	87.1 (1.4)
Urban	12.9 (1.3)	12.9 (1.4)	12.9 (1,4)
**Household income**			
lowest	13.1 (0.6)	11.9 (0.8)	14.4 (0.8)
lower middle	23.5 (0.8)	22.3 (1.0)	24.7 (1.0)
higher middle	29.9 (0.8)	29.9 (1.1)	29.9 (1.0)
highest	33.5 (1.1)	35.9 (1.4)	31.0 (1.3)
**Educational level**			
Middle school or lower	19.8 (0.7)	14.2 (0.8)	25.5 (1.0)
High school	26.6 (0.8)	26.1 (1.2)	27.0 (1.0)
College or more	53.6 (1.0)	59.6 (1.3)	47.5 (1.1)
**High risk alcohol consumption**			
Non-consumer	22.4 (0.7)	14.3 (0.9)	30.6 (1.0)
<1/week	55.9 (0.9)	52.3 (1.3)	59.5 (1.1)
≥1/week	21.7 (0.7)	33.4 (1.2)	9.8 (0.7)
**Smoking status**			
Never	60.7 (0.8)	31.6 (1.2)	90.2 (0.7)
Former	19.7 (0.7)	34.4 (1.2)	4.9 (0.5)
Current	19.5 (0.7)	34.0 (1.2)	4.9 (0.5)
**Physical activity**§			
Yes	52.1 (0.9)	48.6 (1.3)	55.6 (1.1)
No	47.9 (0.9)	51.4 (1.3)	44.4 (1.1)
**Body mass index, kg/m^2^**		
<18.5	3.5 (0.3)	2.5 (0.4)	4.6 (0.5)
18.5–22.9	39.0 (0.8)	30.1 (1.2)	48.1 (1.2)
23–24.9	22.2 (0.7)	25.5 (1.1)	18.9 (0.8)
≥25	35.2 (0.8)	42.0 (1.2)	28.3 (0.8)
**Hs-CRP, mg/dL**			
Mean (SE)	1.13 (0.0)	1.19 (0.0)	1.07 (0.0)
Median (IQR)	0.56 (0.35–1.10)	0.62 (0.40–1.20)	0.50 (0.31–1.00)
**Metabolic variables**			
WC, cm	82.3 (0.2)	86.2 (0.2)	78.4 (0.2)
Triglyceride, mg/dL	137.4 (2.0)	159.6 (3.4)	114.8 (0.4)
Fasting glucose, mg/dL	100.1 (0.4)	102.5 (0.6)	97.8 (0.5)
HDL-cholesterol, mg/dL	51.1 (0.2)	47.4 (0.3)	54.8 (0.3)
SBP, mmHg	117.6 (0.3)	119.9 (0.4)	115.1 (0.4)
DBP, mmHg	75.9 (0.2)	78.3 (0.3)	73.4 (0.2)
**Retinol, mg/L**			
Median (IQR)	0.49 (0.39–0.61)	0.55 (0.45–0.68)	0.44 (0.36–0.55)
**α-tocopherol, mg/L**			
Median (IQR)	12.58 (10.36–15.67)	12.38 (10.07–15.42)	12.76 (10.51–15.84)

Data are presented as weighted percentages (standard error [SE]) for categorical variables or weighted means (SE) and medians (interquartile range [IQR].) for continuous variables, unless otherwise stated. High risk alcohol consumption was defined as 7 (alcohol 60 g) or more drinks for men and 5 (alcohol 40 g) or more drinks for women on occasion. § Adequate physical activity was defined when 1 of 3 following was satisfied: (a) at least 150–300 min per week of moderate intensity, (b) 75–150 min per week of vigorous-intensity physical activity, or (c) equivalent combination of moderate- and vigorous-intensity aerobic activity. Hs-CRP, high sensitivity C-reactive protein; WC, waist circumference; HDL, high density lipoprotein; SBP, systolic blood pressure; DBP, diastolic blood pressure.

**Table 2:** Prevalence and risk of metabolic syndrome according to serum retinol and α-tocopherol levels.

	Prevalence % (SE)	Unadjusted		Adjusted for Age and Sex		Multivariate-Adjusted			
						BMI Unadjusted		Adjusted for BMI	
		OR (95% CI)	*P*_trend_	OR (95% CI)	*P*_trend_	OR (95% CI)	*P*_trend_	OR (95% CI)	*P*_trend_
Retinol			<0.001		<0.001		<0.001		<0.001
Q1	12.1 (1.1)	1		1		1		1	
Q2	20.9 (1.4)	1.912 (1.468–2.491)		1.672 (1.280–2.184)		1.802 (1.368–2.374)		1.703 (1.254–2.313)	
Q3	25.9 (1.5)	2.526 (1.962–3.253)		2.032 (1.561–2.645)		2.243 (1.717–2.931)		2.027 (1.526–2.691)	
Q4	33.5 (1.5)	3.649 (2.868–4.642)		2.651 (2.054–3.423)		2.795 (2.129–3.670)		2.351 (1.748–3.163)	
α-tocopherol			<0.001		<0.001		<0.001		<0.001
Q1	14.5 (1.3)	1		1		1		1	
Q2	18.6 (1.2)	1.338 (1.039–1.723)		1.260 (0.969–1.639)		1.280 (0.977–1.678)		1.302 (0.973–1.743)	
Q3	26.5 (1.5)	2.113 (1.650–2.706)		1.848 (1.436–2.377)		1.910 (1.479–2.466)		1.713 (1.303–2.252)	
Q4	34.9 (1.6)	3.154 (2.503–3.974)		2.643 (2.074–3.368)		2.690 (2.098–3.449)		2.559 (1.953–3.353)	

Serum retinol and α-tocopherol levels were categorized into quartiles; Q1 (lowest), Q2 (lower middle), Q3 (higher middle), and Q4 (highest). Multivariate-adjusted model included age, sex, residence, household income, education, alcohol consumption, smoking status, physical activity, hs-CRP, and BMI (or not) as covariates. *P* for trend was calculated using linear regression model considering serum vitamin levels as continuous variables. Hs-CRP, high sensitivity C-reactive protein; SE, standard error; BMI, body mass index; OR, odds ratio; CI, confidence interval.

**Table 3:** Prevalence (prev.) and risk of components of metabolic syndrome according to serum retinol and α-tocopherol levels.

	Retinol					α-Tocopherol				
	Prev. % (SE)	Multivariate-Adjusted				Prev. % (SE)	Multivariate-Adjusted			
		BMI Unadjusted		Adjusted for BMI			BMI Unadjusted		Adjusted for BMI	
		OR (95% CI)	*P*_trend_	OR (95% CI)	*P*_trend_		OR (95% CI)	*P*_trend_	OR (95% CI)	*P*_trend_
Abdominal obesity			<0.001		0.545			0.001		0.547
Q1	19.9 (1.4)	1		1		24.1 (1.6)	1		1	
Q2	26.3 (1.5)	1.376 (1.085–1.746)		1.111 (0.799–1.545)		25.3 (1.5)	1.048 (0.821–1.339)		0.993 (0.711–1.386)	
Q3	29.5 (1.7)	1.550 (1.223–1.963)		1.161 (0.842–1.603)		29.5 (1.5)	1.253 (0.998–1.573)		0.822 (0.600–1.125)	
Q4	34.2 (1.5)	1.759 (1.386–2.233)		1.125 (0.806–1.570)		32.8 (1.5)	1.384 (1.108–1.730)		0.952 (0.701–1.291)	
High triglycerides			<0.001		<0.001			<0.001		<0.001
Q1	10.8 (1.1)	1		1		13.4 (1.2)	1		1	
Q2	23.4 (1.6)	2.290 (1.718–3.052)		2.149 (1.604–2.878)		21.0 (1.5)	1.948 (1.487–2.551)		1.897 (1.444–2.492)	
Q3	31.7 (1.6)	3.276 (2.495–4.302)		2.980 (2.260–3.929)		33.0 (1.6)	3.941 (3.033–5.122)		3.622 (2.780–4.717)	
Q4	47.3 (1.6)	5.376 (4.054–7.131)		4.718 (3.538–6.293)		49.3 (1.6)	8.149 (6.225–10.668)		7.633 (5.841–9.975)	
Low HDL-cholesterol			0.494		0.465			0.896		0.213
Q1	31.7 (1.6)	1		1		31.3 (1.7)	1		1	
Q2	32.2 (1.7)	1.074 (0.867–1.330)		1.010 (0.811–1.258)		28.2 (1.5)	0.782 (0.629–0.972)		0.748 (0.596–0.938)	
Q3	34.0 (1.5)	1.232 (0.990–1.533)		1.102 (0.878–1.383)		31.7 (1.6)	0.841 (0.683–1.037)		0.762 (0.614–0.944)	
Q4	28.8 (1.5)	1.053 (0.846–1.310)		0.898 (0.715–1.126)		34.5 (1.6)	0.935 (0.755–1.158)		0.832 (0.668–1.037)	
High fasting glucose			<0.001		<0.001			<0.001		0.006
Q1	19.5 (1.3)	1		1		26.0 (1.6)	1		1	
Q2	28.7 (1.5)	1.438 (1.143–1.809)		1.350 (1.067–1.708)		29.0 (1.4)	1.064 (0.838–1.352)		1.031 (0.805–1.320)	
Q3	35.2 (1.7)	1.755 (1.374–2.240)		1.569 (1.221–2.016)		35.8 (1.7)	1.312 (1.028–1.673)		1.168 (0.911–1.497)	
Q4	45.0 (1.7)	2.233 (1.758–2.836)		1.906 (1.489–2.439)		41.2 (1.6)	1.521 (1.198–1.930)		1.353 (1.061–1.726)	
High blood pressure			<0.001		0.007			<0.001		<0.001
Q1	18.4 (1.4)	1		1		21.9 (1.5)	1		1	
Q2	25.8 (1.4)	1.227 (0.952–1.583)		1.162 (0.895–1.508)		25.9 (1.4)	1.149 (0.901–1.466)		1.137 (0.892–1.450)	
Q3	30.8 (1.6)	1.381 (1.067–1.787)		1.272 (0.977–1.655)		32.9 (1.6)	1.468 (1.156–1.863)		1.368 (1.076–1.739)	
Q4	39.5 (1.6)	1.594 (1.233–2.061)		1.420 (1.092–1.847)		36.6 (.1.6)	1.568 (1.244–1.976)		1.460 (1.154–1.846)	

Serum retinol and α-tocopherol levels were categorized into quartiles; Q1 (lowest), Q2 (lower middle), Q3 (higher middle), and Q4 (highest). Multivariate-adjusted model included age, sex, residence, household income, education, alcohol consumption, smoking status, physical activity, hs-CRP, and BMI (or not) as covariates. *P* for trend was calculated using linear regression model considering serum vitamin levels as continuous variables. HDL, high density lipoprotein; hs-CRP, high sensitivity C-reactive protein; SE, standard error; BMI, body mass index; OR, odds ratio; CI, confidence interval.

## Supplementary Materials

The following are available online at https://www.mdpi.com/2072-6643/12/6/1689/s1, Table S1. Unweighted prevalence of metabolic syndrome and its components according to serum retinol and α-tocopherol levels. Table S2. Prevalence and risk of metabolic syndrome according to serum retinol and α-tocopherol levels in men. Table S3. Prevalence and risk of metabolic syndrome according to serum retinol and α-tocopherol levels in women. Table S4. Prevalence and risk of metabolic syndrome according to dietary vitamin A intake (n = 5142). Figure S1. Scatter plot of log-transformed oral vitamin intake (horizontal axis) against log-transformed serum retinol level (vertical axis) in the Korean general population (r = Pearson’s correlation coefficient).
