# Supplementary material for: Association Between Serum Retinol and α-Tocopherol Levels and Metabolic Syndrome in Korean General Population: Analysis of Population-Based Nationally Representative Data
Source: Nutrients. 2020 Jun 5;12(6):1689. doi: 10.3390/nu12061689 (PMC7352386; doi:10.3390/nu12061689)
Supplement: Supplementary file 1 [file nutrients-12-01689-s001.zip › Supplementary Tables 1, 2, 3, 4.docx]

Table S1. Unweighted prevalence of metabolic syndrome and its components according to serum retinol and α-tocopherol level

|  | **Retinol** | | | **α-Tocopherol** | | |
| --- | --- | --- | --- | --- | --- | --- |
|  | Total  N (%) | Men  N (%) | Women  N (%) | Total  N (%) | Men  N (%) | Women  N (%) |
| Metabolic syndrome |  |  |  |  |  |  |
| Q1 | 198 (14.3) | 55 (17.0) | 143 (13.5) | 203 (17.0) | 113 (18.4) | 90 (15.5) |
| Q2 | 306 (22.2) | 129 (25.0) | 177 (20.5) | 286 (19.4) | 163 (24.5) | 123 (15.2) |
| Q3 | 422 (28.6) | 225 (29.7) | 197 (27.6) | 425 (26.9) | 212 (30.6) | 213 (24.0) |
| Q4 | 587 (35.6) | 392 (36.5) | 195 (33.9) | 599 (36.5) | 313 (44.5) | 286 (30.5) |
| Abdominal obesity |  |  |  |  |  |  |
| Q1 | 300 (21.7) | 70 (21.6) | 230 (21.7) | 292 (24.5) | 165 (26.9) | 127 (21.9) |
| Q2 | 369 (26.7) | 173 (33.5) | 196 (22.7) | 383 (26.0) | 207 (31.2) | 176 (21.8) |
| Q3 | 465 (31.6) | 255 (33.6) | 210 (29.4) | 474 (30.0) | 229 (33.1) | 245 (27.6) |
| Q4 | 591 (35.8) | 389 (36.2) | 202 (35.1) | 576 (35.1) | 286 (40.7) | 290 (30.9) |
| High triglyceride |  |  |  |  |  |  |
| Q1 | 172 (12.5) | 66 (20.4) | 106 (10.0) | 162 (13.6) | 117 (19.1) | 45 (7.8) |
| Q2 | 324 (23.5) | 148 (28.7) | 176 (20.4) | 301 (20.4) | 208 (31.3) | 93 (11.5) |
| Q3 | 487 (33.1) | 285 (37.6) | 202 (28.3) | 505 (32.0) | 300 (43.4) | 205 (23.1) |
| Q4 | 783 (47.5) | 580 (54.0) | 203 (35.5) | 798 (48.7) | 454 (64.6) | 344 (36.7) |
| Low HDL-cholesterol |  |  |  |  |  |  |
| Q1 | 462 (33.4) | 104 (32.1) | 358 (33.8) | 408 (34.2) | 166 (27.1) | 242 (41.7) |
| Q2 | 471 (34.1) | 140 (27.1) | 331 (38.3) | 449 (30.5) | 172 (25.9) | 277 (34.3) |
| Q3 | 518 (35.2) | 221 (29.2) | 297 (41.5) | 507 (32.1) | 173 (25.0) | 334 (37.6) |
| Q4 | 487 (29.5) | 254 (23.6) | 233 (40.5) | 574 (35.0) | 208 (29.6) | 366 (39.1) |
| High fasting glucose |  |  |  |  |  |  |
| Q1 | 316 (22.8) | 108 (33.3) | 208 (19.6) | 336 (28.2) | 205 (33.4) | 131 (22.6) |
| Q2 | 418 (30.3) | 193 (37.4) | 225 (26.0) | 460 (31.3) | 258 (38.9) | 202 (25.0) |
| Q3 | 569 (38.6) | 304 (40.1) | 265 (37.1) | 581 (36.8) | 314 (45.4) | 267 (30.1) |
| Q4 | 772 (46.8) | 548 (51.0) | 224 (39.0) | 698 (42.6) | 376 (53.5) | 322 (34.4) |
| High blood pressure |  |  |  |  |  |  |
| Q1 | 260 (18.8) | 83 (25.6) | 177 (16.7) | 262 (22.0) | 178 (29.0) | 84 (14.5) |
| Q2 | 377 (27.3) | 168 (32.6) | 209 (24.2) | 394 (26.8) | 226 (34.0) | 168 (20.8) |
| Q3 | 480 (32.6) | 283 (37.3) | 197 (27.6) | 519 (32.8) | 279 (40.3) | 240 (27.0) |
| Q4 | 669 (40.6) | 457 (42.6) | 212 (36.9) | 611 (37.3) | 308 (43.8) | 303 (32.3) |

Serum retinol and α-tocopherol levels were categorized into quartiles; Q1 (lowest), Q2 (lower middle), Q3 (higher middle), and Q4 (highest).

HDL, high density lipoprotein; N, numbers.

Table S2. Prevalence and risk for metabolic syndrome according to serum retinol and α-tocopherol in men

|  | Prevalence  % (SE) | Unadjusted | | Age adjusted | | Multivariate-adjusted | | | |
| --- | --- | --- | --- | --- | --- | --- | --- | --- | --- |
|  |  |  |  |  |  | BMI unadjusted | | BMI adjusted | |
|  |  | OR (95% CI) | *P* _trend_ | OR (95% CI) | *P* _trend_ | OR (95% CI) | *P* _trend_ | OR (95% CI) | *P* _trend_ |
| Retinol |  |  | <0.001 |  | <0.001 |  | <0.001 |  | 0.003 |
| Q1 | 13.2 (2.3) | 1 |  | 1 |  | 1 |  | 1 |  |
| Q2 | 25.1 (2.5) | 2.205 (1.384-3.512) |  | 2.231 (1.384-3.597) |  | 2.337 (1.457-3.878) |  | 2.064 (1.171-3.641) |  |
| Q3 | 26.0 (2.0) | 2.311 (1.504-3.551) |  | 2.297 (1.479 (3.566) |  | 2.544 (1.625-3.985) |  | 2.157 (1.299-3.581) |  |
| Q4 | 34.0 (1.8) | 3.378 (2.233-5.112) |  | 3.214 (2.097-4.925) |  | 3.073 (1.946-4.852) |  | 2.469 (1.478-4.126) |  |
| α-Tocopherol |  |  | <0.001 |  | <0.001 |  | <0.001 |  | <0.001 |
| Q1 | 16.3 (1.9) | 1 |  | 1 |  | 1 |  | 1 |  |
| Q2 | 23.4 (2.1) | 1.570 (1.109-2.222) |  | 1.466 (1.030-2.087) |  | 1.462 (1.021-2.095) |  | 1.463 (0.986-2.170) |  |
| Q3 | 29.5 (2.2) | 2.156 (1.538-3.021) |  | 1.941 (1.381-2.729) |  | 1.925 (1.360-2.724) |  | 1.684 (1.156-2.454) |  |
| Q4 | 41.3 (2.4) | 3.614 (2.619-4.988) |  | 3.231 (2.332-4.476) |  | 3.063 (2.183-4.298) |  | 2.838 (1.962-4.107) |  |

Serum retinol and α-tocopherol levels were categorized into quartiles; Q1 (lowest), Q2 (lower middle), Q3 (higher middle), and Q4 (highest).

Multivariate-adjusted model included age, sex, residence, household income, education, alcohol consumption, smoking status, physical activity, hs-CRP, and BMI (or not) as covariates.

*P* for trend was calculated using linear regression model considering serum vitamin levels as continuous variables.

SE, standard error; BMI, body mass index; OR, odds ratio; CI, confidence interval.

Table S3. Prevalence and risk for metabolic syndrome according to serum retinol and α-tocopherol in women

|  | Prevalence  % (SE) | Unadjusted | | Age adjusted | | Multivariate-adjusted | | | |
| --- | --- | --- | --- | --- | --- | --- | --- | --- | --- |
|  |  |  |  |  |  | BMI unadjusted | | BMI adjusted | |
|  |  | OR (95% CI) | *P* _trend_ | OR (95% CI) | *P* _trend_ | OR (95% CI) | *P* _trend_ | OR (95% CI) | *P* _trend_ |
| Retinol |  |  | <0.001 |  | <0.001 |  | <0.001 |  | <0.001 |
| Q1 | 11.7 (1.2) | 1 |  | 1 |  | 1 |  | 1 |  |
| Q2 | 17.7 (1.6) | 1.612 (1.174-2.214) |  | 1.302 (0.938-1.806) |  | 1.444 (1.028-2.029) |  | 1.458 (1.022-2.081) |  |
| Q3 | 25.7 (2.0) | 2.601 (1.924-3.516) |  | 1.795 (1.292-2.495) |  | 2.053 (1.453-2.901) |  | 2.029 (1.432-2.876) |  |
| Q4 | 32.6 (2.5) | 3.630 (2.659-4.957) |  | 2.038 (1.453-2.860) |  | 2.355 (1.647-3.368) |  | 2.251 (1.512-3.351) |  |
| α-Tocopherol |  |  | <0.001 |  | <0.001 |  | <0.001 |  | <0.001 |
| Q1 | 12.1 (1.6) | 1 |  | 1 |  | 1 |  | 1 |  |
| Q2 | 13.7 (1.4) | 1.148 (0.791-1.665) |  | 0.940 (0.637-1.388) |  | 1.027 (0.684-1.543) |  | 1.084 (0.703-1.672) |  |
| Q3 | 23.7 (1.8) | 2.248 (1.554-3.252) |  | 1.504 (1.044-2.167) |  | 1.662 (1.139-2.425) |  | 1.614 (1.060-2.459) |  |
| Q4 | 29.2 (2.0) | 2.988 (2.119-4.213) |  | 1.768 (1.237-2.526) |  | 1.904 (1.316-2.754) |  | 2.035 (1.342-3.087) |  |

Serum retinol and α-tocopherol levels were categorized into quartiles; Q1 (lowest), Q2 (lower middle), Q3 (higher middle), and Q4 (highest).

Multivariate-adjusted model included age, sex, residence, household income, education, alcohol consumption, smoking status, physical activity, hs-CRP, and BMI (or not) as covariates.

*P* for trend was calculated using linear regression model considering serum vitamin levels as continuous variables.

SE, standard error; BMI, body mass index; OR, odds ratio; CI, confidence interval.

**Table S4.** Prevalence and risk of metabolic syndrome according to dietary vitamin A intake (n=5142).

|  | Prevalence  % (SE) | Unadjusted | | Age and (sex) adjusted | | Multivariate-adjusted | | | |
| --- | --- | --- | --- | --- | --- | --- | --- | --- | --- |
|  |  |  |  |  |  | BMI unadjusted | | BMI adjusted | |
|  |  | OR (95% CI) | *P* _trend_ | OR (95% CI) | *P* _trend_ | OR (95% CI) | *P* _trend_ | OR (95% CI) | *P* _trend_ |
| Total |  |  | 0.137 |  | 0.080 |  | 0.008 |  | 0.074 |
| Q1 | 23.6 (1.4) | 1 |  | 1 |  | 1 |  | 1 |  |
| Q2 | 22.4 (1.4) | 0.936 (0.749-1.171) |  | 1.072 (0.846-1.358) |  | 1.139 (0.894-1.451) |  | 1.121 (0.859-1.462) |  |
| Q3 | 25.1 (1.4) | 1.085 (0.875-1.345) |  | 1.182 (0.939-1.488) |  | 1.305 (1.032-1.649) |  | 1.304 (1.011-1.682) |  |
| Q4 | 25.8 (1.5) | 1.128 (0.915-1.390) |  | 1.194 (0.957-1.490) |  | 1.331 (1.058-1.675) |  | 1.227 (0.944-1.596) |  |
| Men |  |  | 0.095 |  | 0.029 |  | 0.021 |  | 0.113 |
| Q1 | 24.5 (2.2) | 1 |  | 1 |  | 1 |  | 1 |  |
| Q2 | 28.1 (2.3) | 1.202 (0.873-1.655) |  | 1.354 (0.977-1.878) |  | 1.385 (0.991-1.935) |  | 1.400 (0.968-2.026) |  |
| Q3 | 27.1 (2.2) | 1.145 (0.831-1.579) |  | 1.277 (0.919-1.774) |  | 1.293 (0.925-1.807) |  | 1.306 (0.898-1.899) |  |
| Q4 | 30.4 (2.1) | 1.346 (0.998-1.815) |  | 1.495 (1.102-2.028) |  | 1.540 (1.129-2.102) |  | 1.434 (1.001-2.055) |  |
| Women |  |  | 0.552 |  | 0.988 |  | 0.288 |  | 0.375 |
| Q1 | 22.9 (1.8) | 1 |  | 1 |  | 1 |  | 1 |  |
| Q2 | 17.1 (1.6) | 0.697 (0.515-0.944) |  | 0.850 (0.613-1.178) |  | 0.933 (0.664-1.311) |  | 0.893 (0.619-1.288) |  |
| Q3 | 22.9 (1.8) | 0.999 (0.750-1.331) |  | 1.132 (0.827-1.550) |  | 1.358 (0.976-1.890) |  | 1.377 (0.974-1.949) |  |
| Q4 | 19.4 (2.0) | 0.809 (0.592-1.107) |  | 0.898 (0.643-1.254) |  | 1.056 (0.743-1.501) |  | 1.020 (0.686-1.517) |  |

Dietary vitamin A intake was categorized into quartiles; Q1 (lowest), Q2 (lower middle), Q3 (higher middle), and Q4 (highest).

Intake amount of dietary vitamin A was measured with the use of Retinol Equivalents (RE, mcg RE = mcg retinol + mcg carotenes/6).

Multivariate-adjusted model included age, sex, residence, household income, education, alcohol consumption, smoking status, physical activity, hs-CRP, and BMI (or not) as covariates.

*P* for trend was calculated using linear regression model considering dietary vitamin A intake as continuous variables.

SE, standard error; hs-CRP; high sensitivity C-reactive protein; BMI, body mass index; OR, odds ratio; CI, confidence interval.
