# Supplementary figures and images for: Association Between Serum Retinol and α-Tocopherol Levels and Metabolic Syndrome in Korean General Population: Analysis of Population-Based Nationally Representative Data
Source: Nutrients. 2020 Jun 5;12(6):1689. doi: 10.3390/nu12061689 (PMC7352386; doi:10.3390/nu12061689)

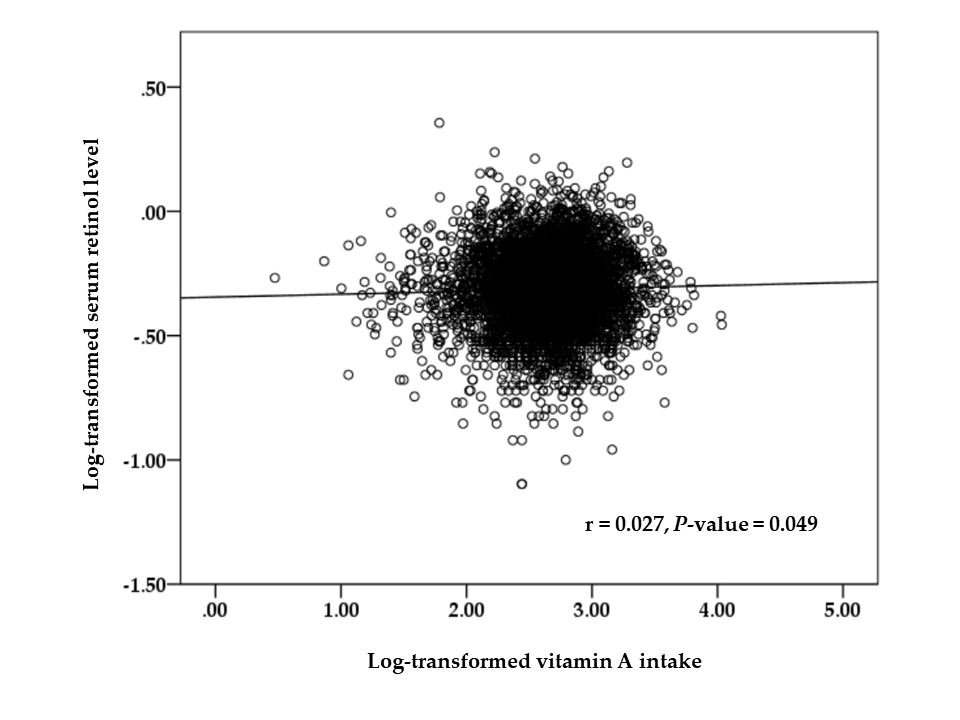

Supplement: Supplementary file 1 [file nutrients-12-01689-s001.zip › Figure S1.tif]
